# Clinical versus histological grading in the assessment of cutaneous graft versus host disease

**DOI:** 10.1186/s40001-019-0377-6

**Published:** 2019-04-10

**Authors:** M. C. H. Hogenes, L. C. J. te Boome, D. C. van der Valk, M. R. van Dijk, R. A. de Weger, J. Kuball, P. J. van Diest

**Affiliations:** 1 LABPON, Laboratory for Pathology East Netherlands, Boerhaavelaan 59, P.O. Box 510, 7550 AM Hengelo, The Netherlands; 2 MCH Haaglanden Department Internal Medicine, The Hague, The Netherlands; 3 Department of Hematology, UMC Utrecht, Utrecht, The Netherlands; 4 Department of Pathology, University Medical Centre Utrecht, PO box 85500, 3508 GA Utrecht, The Netherlands; 5 Laboratory of Translational Immunology, UMC Utrecht, Utrecht, The Netherlands

**Keywords:** Graft versus host disease, Skin diseases, Histology, Grading, Prognosis

## Abstract

**Background:**

Skin biopsies are often used in daily practice for the diagnosis of acute (aGvHD) or chronic graft versus host disease (cGvHD). With the latest understanding in pathogenesis and new National Institute of Health (NIH) classifications for aGvHD and cGvHD, there is a need to evaluate the current prognostic value of histological grading cutaneous GvHD and its correlation to the clinical grade.

**Methods:**

In a retrospective study with 120 skin biopsies (all taken for suspected GvHD) from 110 patients (all classified according to the NIH), biopsies were revised and graded, blinded for clinical information, for either acute of chronic features. Morphological grades were compared for concordance with the clinical grade and survival analyses were done for clinical and histological grading.

**Results:**

Correlation for histologic vs. clinical grading was (very) poor for aGvHD and cGvHD (weighted *κ* − 0.038 and 0.0009, respectively). Patients with clinical aGvHD had worse prognosis compared to cGvHD. However, at time of biopsy neither clinical nor histological grading predicted the eventual survival for either aGvHD (*p* = 0.9739 and *p* = 0.0744, respectively) or cGvHD (*p* = 0.2149 and *p* = 0.4465, respectively).

**Conclusions:**

Confirming the diagnosis of GvHD is still a valuable reason for taking a skin biopsy, but this study shows that histologic grading of GvHD in the skin biopsy has no additional value for clinicians in current practice.

**Electronic supplementary material:**

The online version of this article (10.1186/s40001-019-0377-6) contains supplementary material, which is available to authorized users.

## Introduction

Allogeneic hematopoietic cell transplantation (HCT) is a potentially curative therapy with proven efficacy in the management of hematologic malignancies. However, it can be complicated by the syndromes of acute and chronic graft versus host disease. Graft versus host disease (GvHD) is a major cause of morbidity and mortality of HCT. Skin, liver, and intestine are regarded as the principal target organs of GvHD and can be affected to varying degrees or not at all.

Skin biopsies are often taken to differentiate cutaneous GvHD from other diseases with similar cutaneous symptoms. Although some reports question the value of skin biopsies since histological features may overlap with other skin diseases, they are by most still regarded essential in diagnosing aGvHD. For cGvHD their value is less clear.

To assess severity of cutaneous GvHD, several grading systems have been developed. These either have a clinical or histological point of view.

Clinically cutaneous cGvHD is either sub-classified as limited or extended [1] or as mild, moderate, or severe cGvHD [2, 3]. New classification systems incorporate clinical symptoms and patient’s functional status, but sometimes fail to include lichenoid features separately and mainly focus on sclerosis [2] or do not differentiate between both types within their grading system [3]. However, lichenoid and sclerotic cGvHD show different responses to treatment, in which lichenoid cGvHD has a poor prognosis [4, 5].

In the histological evaluation of a skin biopsy, pathologists try to assist clinical decision makers by confirming the diagnosis GvHD and grade the morphological severity. The histologic criteria for diagnosing cutaneous GvHD include features for aGvHD as well as lichenoid and sclerotic cGvHD patterns [6]. For aGvHD, Horn’s adapted Lerner grading system [7, 8] is often used. For cGvHD however, there are no strictly formulated histological criteria to evaluate severity of either lichenoid or sclerotic cGvHD.

Correlation studies between clinical and histological features have been performed previously for gastrointestinal biopsies [9–12] in which a correlation between clinical and histological grading has both been suggested [11] as well as denied [12]. In cutaneous GvHD, a similar lack of correlation between clinical and histological grading has been reported [13], but that was long before the clinical manifestations and new classifications were taken into account.

In this study, we therefore analyzed the current prognostic value of skin biopsies in GvHD and whether histological grading of acute and chronic GvHD correlates with clinical manifestations.

## Material and method

### Patients’ selection and clinical data

According to the JACIE and EBMT guidelines, transplantation data were collected from patients and donors after informed consent was signed in the allogeneic HCT unit of the Hematology Department of the University Medical Centre of Utrecht (UMCU). A search was performed in the local pathology database for patients diagnosed with GvHD based on a skin biopsy between April 2007 and September 2011. Cases with more than 1 skin biopsy during follow-up were treated as single individuals (*n* = 110) for patient-related statistics, and the skin biopsies were considered separate events in our analyses with histology (*n* = 120). Patients were clinically classified according to the NIH definitions [6] as aGvHD (including classical aGvHD and persistent, recurrent or late onset aGvHD, after HCT or donor lymphocyte infusion) or cGvHD (including classical cGvHD—chronic progressive, quiescent, or de novo- and overlap syndromes of cGvHD with aGvHD). Clinical grading of GvHD was done following the adapted grading system from Glucksberg [14] and Pzerpiorka [15] for aGvHD and the Seattle criteria proposed by Shulman et al. [1] were used to distinguish limited and extensive cGvHD (Table 1). In order to evaluate the percentage of skin involvement in cGvHD, percentages were chosen that are similar to the acute criteria. A summary of the baseline statistics of our patients can be found in Table 3.

**Table 1:** Clinical grading system for acute and chronic graft versus host disease

Clinical grading acute GvHD^a^				Total clinical grade acute GvHD^a^					Clinical grading chronic GvHD^c^	
Grade per organ	Involvement skin: % body surface area	Involvement GI: volume diarrhea (ml/day)	Involvement liver: level bilirubin (μmol/l)	Total grade aGvHD	Skin grade	GI grade	Liver grade	Clinical performance	Grade	Criteria
0	No rash caused by GvHD	< 500	< 20	0	0	0	0	Normal	Limited	Only involvement of skin and liver
1	< 25%	500–1000; or nausea and vomiting with positive biopsy	20–40; with moderate increase in ASAT (150–170 IU)	1	1–2	0	0	Normal	Extensive	Involvement of skin, liver, and any other organ
2	25–50%	1000–1500; or more severe nausea and vomiting	40–75	2^b^	1–3	1	1	Mildly decreased		
3	> 50%	1500–2000; or more severe nausea and vomiting	75–200	3^b^	2–3	2–3	2–4	Markedly decreased		
4	> 50% with bullous formation or desquamation	> 2000; or severe abdominal pain with or without ileus	> 200	4	2–4	2–4	2–4	Extremely decreased		

^a^Grading adapted from Glucksberg et al. [14] and Przepiorka et al. [15]

^b^Grades 2 and 3 must involve GI and liver at indicated organ grades, in addition to skin involvement

^c^Grading according to the Seattle criteria by Shulman et al. [1]

Revision of the clinical grading (for skin involvement as well as overall clinical grading) was performed based on the actual documented clinical symptoms and signs and compared to the original clinical grading for coherence.

### Histological grading

All skin biopsies retrieved in this search were revised by two pathologists and graded blinded to clinical information for lichenoid (as a representation of acute/active) and sclerotic (as a representation of chronic) morphological features. The pathologists were unaware of the interval between biopsy and transplantation as well as the clinical symptoms and had to decide for acute and chronic features purely based on morphology of the skin biopsy. Revised grades were compared to the original grading at time of biopsy for coherence.

For the lichenoid/acute grading, Horn’s criteria [7] were adapted (Table 2). Due to current lack of official grading systems for sclerotic/chronic GvHD, we have developed a grading system focusing on limited or extensive sclerotic changes for the purpose of this study (Table 2). The hypothesis used is that severity of sclerosis can be most objectively reflected as the extent of sclerosis into the deeper dermis, often resulting in loss of sub-epidermal fat and incorporation of adnexal structures of the skin.

**Table 2:** Histological grading system for acute and chronic cutaneous graft versus host disease

Histological grading of acute GvHD^a^	
Grade	Morphological criteria
0	Normal skin or epidermal changes due to other causes than GvHD
1	Vacuolar alteration of junction between epidermis and dermis
2	Grade 1 with dyskeratotic cells within the epidermis and/or hair follicle, infiltrate of lymphocytes within the dermis
3	Grade 2 with fusion of vacuoles to form clefts and microvesicles
4	Separation of epidermis from dermis

Histological grading chronic GvHD (proposed criteria focusing on sclerotic features only)^b^	
Grade	Criteria
Limited	Sclerosis within the superficial dermis, including coarsening of the fibers
Extensive	Sclerosis extending into the deep dermis or very dense sclerosis in any layer of the skin

^a^According to Horn’s adapted criteria from Lerner [7]

^b^In histological grading, the lichenoid variant of chronic GvHD was graded according to the components in acute GvHD

### Statistics

Coherence between clinical and histological grading was performed using kappa, Weighted kappa, and correlation between histology and clinical grading using Pearson’s *r* test. Survival analyses were done by plotting Kaplan–Meier curves and the Log Rank test. All tests were done with Graphpad Prism version 5.0. *p* values below 0.05 were regarded significant.

## Results

Results of our analysis are summarized in Table 3 (for patient statistics), Table 4 (for biopsy statistics), and Table 5 (comparison and correlation statistics). For each analysis in our patients and biopsy analysis, the number of available cases for evaluation from our data collection is provided.

**Table 3:** Patients’ statistics (with survival comparison), each patient as single individual

Patient statistics (with survival comparison)				
Feature	Grouping/specifics	Frequency	%	*p* value
Number of patients	*N*	110	100.0%	
Survival patients overall (years)	Mean	2.96 (SD 2.47)		
Gender	Male	72	65.5%	0.526
	Female	38	34.5%	
Age	Mean	45.9 (SD 17.5)		
	0–10	8	7.3%	0.328
	10–20	4	3.6%	
	20–30	8	7.3%	
	30–40	11	10.0%	
	40–50	19	17.3%	
	50–60	36	32.7%	
	60–70	24	21.8%	
Disease	Lymphoma	18	16.4%	0.3137
	Plasma cell disorders	23	20.9%	
	MDS/MPN	12	10.9%	
	Bone marrow failure	3	2.7%	
	Inherited disorders	6	5.5%	
	Metabolic disorders	1	0.9%	
	AML	25	22.7%	
	CML	3	2.7%	
	ALL	11	10.0%	
	CLL	8	7.3%	
Donor type	Sibling (SIB)	34	30.9%	0.1867
	Cord blood (CB)	11	10.0%	
	Matched unrelated donor (MUD)	63	57.3%	
	Other	2	1.8%	
Conditioning type	Myeloablative	17	15.5%	0.2449
	Non-myeloablative	72	65.5%	
	Reduced intensity	21	19.1%	
Infection after treatment	Overall	95	86.4%	0.1582
	CMV	32	29.1%	0.9485
	HHV6	10	9.1%	0.2073
	EBV	7	6.4%	0.6031
	Aspergillus	11	10.0%	0.2082
	Candida	31	28.2%	0.2318
Clinical GvHD	Overall (fully documented) including acute GvHD only (a), both acute and chronic GvHD (b) and chronic GvHD (c)	108	98.2%	0.0037
	Acute GvHD only (a)	18	16.4%	
	Both acute and chronic GvHD (b)	77	70.0%	
	Chronic GvHD only (c)	13	11.8%	
Clinical acute GvHD (a+b)	Mean survival (years)	3.0 (SD 2.6)		
	Overall no. of patients with documented acute GvHD (a+b)	95	86.4%	
	aGvHD presentation at date skin biopsy (with no. of biopsies)	67 (68)	60.9%	0.0036
	Max. clinical documented aGvHD skin grade reached	66		< 0.0001
Clinical chronic GvHD (b+c)	Mean survival (years)	3.3 (SD 2.5)		
	Overall no. of patients with documented chronic GvHD (b+c)	90	81.8%	
	cGvHD presentation at date biopsy (with no. of biopsies)	48 (52)	43.6%	0.44
	Progressive	13 (15)		
	Quiescent	6 (7)		
	Late onset aGvHD	19 (20)		
	De Novo	4 (4)		
	Late onset aGvHD after DLI	6 (6)		
	Max. clinical documented cGvHD grade reached (including all tracts)	48	43.6%	0.8844
	Max. clinical documented % skin involvement	46	41.8%	0.7622
	0%	6		
	0–25%	15		
	25–50%	25		
	> 50%	0

**Table 4:** Patients’ statistics (with survival comparison), each biopsy being a single event

Biopsy statistics (with survival comparison)				
Feature	Grouping/specifics	Frequency	%	*p* value
Number of biopsies	*N*	120	100.0%	
Clinical GvHD				
Overall (not aGvHD or cGvHD specified)	Graded based on % skin involvement	117	97.5%	1.668
	No GvHD (0%)	1		
	Grade 1 (< 25%)	24		
	Grade 2 (25–50%)	57		
	Grade 3 (> 50%)	35		
	Grade based on involved tracts	118		0.4059
	Limited (only skin and liver)	67		
	Extensive (involvement of skin, liver, and any other organ)	51		
Clinical acute GvHD at date biopsy	Overall grade (all tracts included)	66	55.0%	0.6812
	No aGvHD	1		
	Grade 1	10		
	Grade 2	33		
	Grade 3	23		
	Grade 4	0		
	Grade skin	66	55.0%	0.7702
	No aGvHD	0		
	Grade 1	7		
	Grade 2	59		
	Grade 3	24		
	Grade 4	0		
Clinical chronic GvHD at date biopsy	Overall grade (all tracts included)	48	40.0%	0.1062
	Limited	15		
	Extensive	33		
	“Grade” skin (% skin involvement)	48	40.0%	
	No skin involvement	0		0.5231
	< 50% skin involved (limited)	35		
	> 50% skin involved (extensive)	13		
Histological GvHD				
Histological acute GvHD in biopsy	Histological acute grade (Horn)	117	97.5%	0.9509
	No aGvHD	8		
	Grade 1	47		
	Grade 2	54		
	Grade 3	8		
Histological chronic GvHD in biopsy	Histological chronic/sclerosis grade	115	95.8%	0.829
	No sclerosis	10		
	Limited	30		
	Extensive	75		
Biopsies of patients presenting with clinical aGvHD	Histological acute grade (Horn)	66	55.0%	0.8111
	No aGvHD	3		
	Grade 1	25		
	Grade 2	34		
	Grade 3	4		
	Histological chronic/sclerosis grade	64	53.3%	0.5068
	No sclerosis	7		
	Limited	16		
	Extensive	41		
Biopsies of patients presenting with clinical cGvHD	Histological acute grade (horn)	46	38.3%	0.8306
	No aGvHD	5		
	Grade 1	20		
	Grade 2	17		
	Grade 3	4		
	Histological chronic/sclerosis grade	46	38.3%	0.7996
	No sclerosis	3		
	Limited	14		
	Extensive	29

**Table 5:** General statistics with kappa and correlation evaluation between clinical and histological grading of acute and chronic (cutaneous) GvHD

General statistics				
Feature				
Number of biopsies:			*N* = 120	
Kappa score evaluation	Comparing features		Kappa	Weighted kappa
Histology versus clinical	Revised histological aGvHD	Revised clinical aGvHD	0.014 (poor)	− 0.038 (very poor)
	Revised histological cGvHD	Revised clinical cGvHD	0.015 (poor)	0.0009 (poor)
Histology	Revised histological aGvHD	Original histological aGvHD grade	0.353 (fair)	0.391 (fair)
	Revised histological cGvHD	Original histological cGvHD grade	Not possible (no original grades)	
Clinical	Revised clinical aGvHD	Original clinical aGvHD grade	0.526 (moderate)	0.489 (moderate)
	Revised clinical cGvHD (all tracts)	Original clinical cGvHD grade (all tracts)	0.909 (very good)	0.829 (very good)

Correlation evaluation	Comparing features		*p* value		
Biopsies from all patients	Revised histological score aGvHD	Revised clinical aGvHD	0.6364		
	Revised histological score cGvHD	Revised clinical cGvHD overall	0.7843		
	Revised histological score cGvHD	Revised max. clinical cGvHD score skin	0.2382		
		Limited 0–50% skin involvement			
		Extensive > 50% skin involvement			
Biopsies from aGvHD patients	Revised histological aGvHD	Revised clinical aGvHD	0.3409		
	Revised histological aGvHD	Max. documented clinical aGvHD	0.3498		
	Revised histological aGvHD	Survival	0.0744		
	Revised clinical GvHD	Survival	0.9739		
Biopsies from cGvHD patients	Revised histological cGvHD	Revised clinical cGvHD overall	0.5105		
	Revised histological cGvHD	Revised clinical cGvHD skin	0.157		
		Limited 0–50% skin involvement			
		Extensive > 50% skin involvement			
	Revised histological cGvHD	Revised max. clinical cGvHD score skin	0.157		
		Limited 0–50% skin involvement			
		Extensive > 50% skin involvement			
	Revised histological cGvHD	Survival	0.4465		
	Revised clinical cGvHD overall	Survival	0.0002		
	Revised max. clinical cGvHD score skin	Survival	0.1249		
	Limited 0–50% skin involvement				
	Extensive > 50% skin involvement				

### Evaluation of patients

Table 3 shows that in 110 patients, mean survival was 2.96 years (SD 2.47), without significant effects on survival of gender, age range, disease, donor type, conditioning regimen and without a significant effect of infection after transplantation (which occurred in a high number of patients). Considering clinical GvHD, patient files were found to be fully documented for our topics of interest in 98.2% of our individuals, revealing that patients either were known clinically with aGvHD only, had both aGvHD as well as cGvHD, or experienced a form of cGvHD without a preceding aGvHD. Survival between these groups was significantly different (*p* = 0.0037). Separating these patients in more rigid groups (just acute and chronic GvHD, without considering the potential overlap of experiencing both at some time during follow-up), 86.4% of patients had a documented form of aGvHD, of which 60.9% presented themselves having aGvHD when their skin biopsy was taken. Presenting clinically with an acute form of GvHD had a significant negative effect on survival, when compared to presenting clinically with cGvHD (*p* = − 0.0036) and the clinical maximum skin grade (the maximum percentage of skin involved in these patients) also showed significant differences in survival (*p* < 0.0001).

81.4% of our patients were known with a documented chronic form of clinical GvHD, but the type of cGvHD presentation (based on the NIH classification) showed no differences in survival (*p* = 0.44). The maximum overall clinical grade of cGvHD reached had no significant effect (*p* = 0.8844) either, nor did the percentage of skin involved when looking at skin only (*p* = 0.7622). Even though clinical cGvHD in patients did not show significant differences in survival between the different forms of cGvHD, the clinical grade of cGvHD did correlate with survival (*p* = 0.0002).

### Evaluation of biopsies

When all skin biopsies were considered a separate event (*n* = 120), survival did not significantly differ with the percentage of skin involved at time of biopsy (*p* = 1.668). However, survival was not significantly different when considering the overall acute clinical grade including all tracts involved either (*p* = 0.4059). For patients presenting clinically as aGvHD at the time of biopsy, there was no effect of the skin grade (*p* = 0.7702) or overall clinical acute grade on survival (*p* = 0.6812). For patients with cGvHD at the time of skin biopsy, the overall clinical grade had no significant effect (*p* = 0.1062). Even though the Seattle criteria normally do not use severity of each tract involved, we also evaluated the effect of skin percentage involved in these cases (as this is used when grading for aGvHD), but this did not influence survival either (*p* = 0.5231).

Both patients with clinical aGVHD and clinically cGvHD at time of biopsy could show histological features of acute and chronic/sclerotic histological GvHD. There is no significant effect on survival for either patients with aGvHD (*p* = 0.8111), nor for those with clinical cGvHD (*p* = 0.7996) at the presentation of their skin biopsy (Additional file 1: Figure S1 and Additional file 2: Figure S2), nor for the histological grading at that time (Additional file 1: Figure S1 and Additional file 2: Figure S2). In fact, there was no effect on survival at all when purely looking at acute histological features or chronic (sclerotic) features when comparing survival based on histological criteria only either (histological aGvHD *n* = 117, *p* = 0.9509 and histological chronic/sclerotic features (*n* = 115, *p* = 0.829).

Comparing kappa scores for revised histological versus clinical grading, correlation was very poor for aGvHD (*κ* 0.014), even when adjusting for the degree of difference in grade (weighted *κ* − 0.038) and poor for cGvHD (*κ* 0.015, weighted *κ* 0.0009). There was no significant correlation between histological scoring and clinical scoring of both aGvHD as well as cGvHD (Table 5 general statistics). Interobserver variability between both pathologists at revision of histology was very low for acute GvHD (*κ* 0.090 and weighted *κ* 0.945) as well as chronic GvHD (*κ* 0.960 and weighted *κ* 0.968) and complete consensus for each histological grade was reached after consultation.

## Discussion

The aim of this study was to evaluate the value of histological grading of cutaneous GvHD and to compare its correlation to the clinical grading of cutaneous GvHD. In summary, our results confirm the lack of correlation between histological and clinical grading of both acute as well as chronic GvHD. For survival, all that seems to matter is the presence of clinical aGvHD and the percentage of skin involved when having aGvHD in skin, while histologic grading of acute and chronic GvHD at the time of biopsy had no prognostic value. The results, however, should be regarded with caution, for several reasons.

First, as GvHD is a multi-organ disease, predicting survival on cutaneous GvHD alone does not reflect the importance of GvHD symptoms in other involved tracts for a patient’s prognosis. This could explain the lack of prognostic value for histological grading of a skin biopsy only.

Second, as our cases were selected based on having a skin biopsy with GvHD, we do have certain selection bias in our study. Most of these skin biopsies will only have been taken when diagnosing cutaneous GvHD was clinically difficult. The small number of histologically grade III aGvHD in our study can be considered a reflection of this issue. Biopsies could have been taken early in the development of the disease and grade might progress after the biopsy was taken. We cannot be sure that histological grading of skin biopsies makes no sense whatsoever if a skin biopsy would be taken *at all* times. However, in our current daily practice in which biopsies are usually just performed to confirm clinical suspicion early and need to be justified in view of costs and morbidity, grading GvHD in a biopsy to predict survival has no value at this moment.

Third, treatment (after histologically establishing GvHD) has a considerable beneficial effect on survival of the individual GvHD patient which may obscure the natural adverse course of histological high-grade GvHD.

Last, grading chronicity of GvHD using sclerosis is rather complex due the lack of existing grading systems for cGvHD, but also confounding other causes for sclerosis.

To address the grading issue for cGvHD, we proposed a grading system using the extension of sclerosis to the deeper dermis as a reflection of severity. This might not fully reflect the natural progression of a cutaneous cGvHD, but in our opinion is the most objective way to evaluate sclerosis. Unfortunately, deeper dermal involvement might be missed when a biopsy was too superficial. That might have influenced the results of our analysis.

We noticed that in many cases of clinical aGvHD, the biopsies often already morphologically showed a certain degree of sclerosis. It is possible that in daily practice this fact is overlooked and therefore not recognized or mentioned in reports. A plausible hypothesis, apart from co-existence of delayed aGvHD in patients with existing sclerodermatous cGvHD or a combination of clinically both lichenoid and sclerotic cGvHD, is that sclerosis histologically might develop in patients with aGvHD even before clinical symptoms of cGvHD occur. However, confounding causes for sclerosis cannot be excluded. A possible effect of immunosuppressive treatment in the formation of dermal fibrosis has been previously addressed by Shulman et al. [6]. Their report also addresses the difficult overlapping features between active cGvHD and aGvHD that might influence the interpretation of a skin biopsy as well as the risk of false-negative results when a biopsy is taken too soon after developing symptoms. It also refers to false-positive results due to recurrent infections, drug reactions, or other inflammatory reactions, although not all infections will influence survival [16]. In other words, the presence of sclerosis in a skin biopsy is subject to many causes and, therefore, using sclerosis to grade cGvHD remains difficult.

The design of our study underscores the importance of clinical information at time of biopsy. In our study, the skin biopsies were histologically graded blinded to clinical information, and therefore the interpretation was susceptible for false-positive or false-negative results. Skin biopsies diagnosed as aGvHD in our study might in fact have been active cGvHD cases. In addition, the presence of collagen in the dermis is subject to the biopsy location, so lack of information on biopsy site might influence the grading of the sclerodermatous components [6]. We feel that by referring these cases as having aGvHD features in our study, the activity and possible implications to the clinical features compared to a more sclerotic reaction are still properly addressed. Nevertheless, in our study neither acute nor chronic histological features appeared to influence patient survival.

Our study was retrospective and we noticed that in the original grading of the disease both clinically as well as histologically, criteria were not always used correctly. This reflects the rather cumbersome and difficult staging and grading criteria, which are frequently not accurately followed outside clinical trials. Our revised data correlate very well for clinical cGvHD cases, moderately for clinical aGvHD cases, but only fairly for aGvHD features. In order to tackle this issue, our evaluation was based on the documented clinical features to revise clinical grade and a blinded revision of histology. Performing a prospective study in which both clinicians and pathologists are restricted to official grading standards (and perhaps applying double reading) is nevertheless highly recommended to confirm our current results.

In conclusion, we feel that at present skin biopsies in daily practice serve no other purpose than to confirm or deny the clinical diagnosis GvHD when in doubt. As the histologically acute features and sclerotic features do not restrict themselves to an acute or chronic group of patients and the presence of cutaneous GvHD alone does not reflect the importance of other involved organs systems either for the survival of a patient, we recommend to stop classifying the histological findings in a skin biopsy into acute or chronic in our pathology reports and just lump the histological diagnosis into one group of ‘histological findings consistent with GvHD’ with either lichenoid or sclerodermatous features, leaving the sub-classification into acute or chronic presentation to our clinicians. Histological grading of a skin biopsy on its own does not predict a patient’s survival and should be used either with lots of caution or preferably not at all to avoid incorrect assumptions on prognosis.

## Additional files

**Additional file 1: Figure S1.** Survival proportions of acute cutaneous GvHD in patients presenting as acute GvHD at time biopsy (n = 66). A. Survival comparison of acute clinical cutaneous GvHD grading (p = 0.6812). B. Survival comparison of histologically acute GvHD grade in skin biopsies (p = 0.8111).

**Additional file 2: Figure S2.** Survival proportions of chronic cutaneous GvHD in patients presenting as chronic GvHD at time of biopsy (n = 48). A. Survival comparison between clinical percentage of skin involved (p = 0.5231). B. Survival comparison of histologically chronic GvHD grade in skin biopsies (p = 0.7996).
