# Supplementary figures and images for: Clinical versus histological grading in the assessment of cutaneous graft versus host disease
Source: Eur J Med Res. 2019 Apr 10;24:19. doi: 10.1186/s40001-019-0377-6 (PMC6457000; doi:10.1186/s40001-019-0377-6)

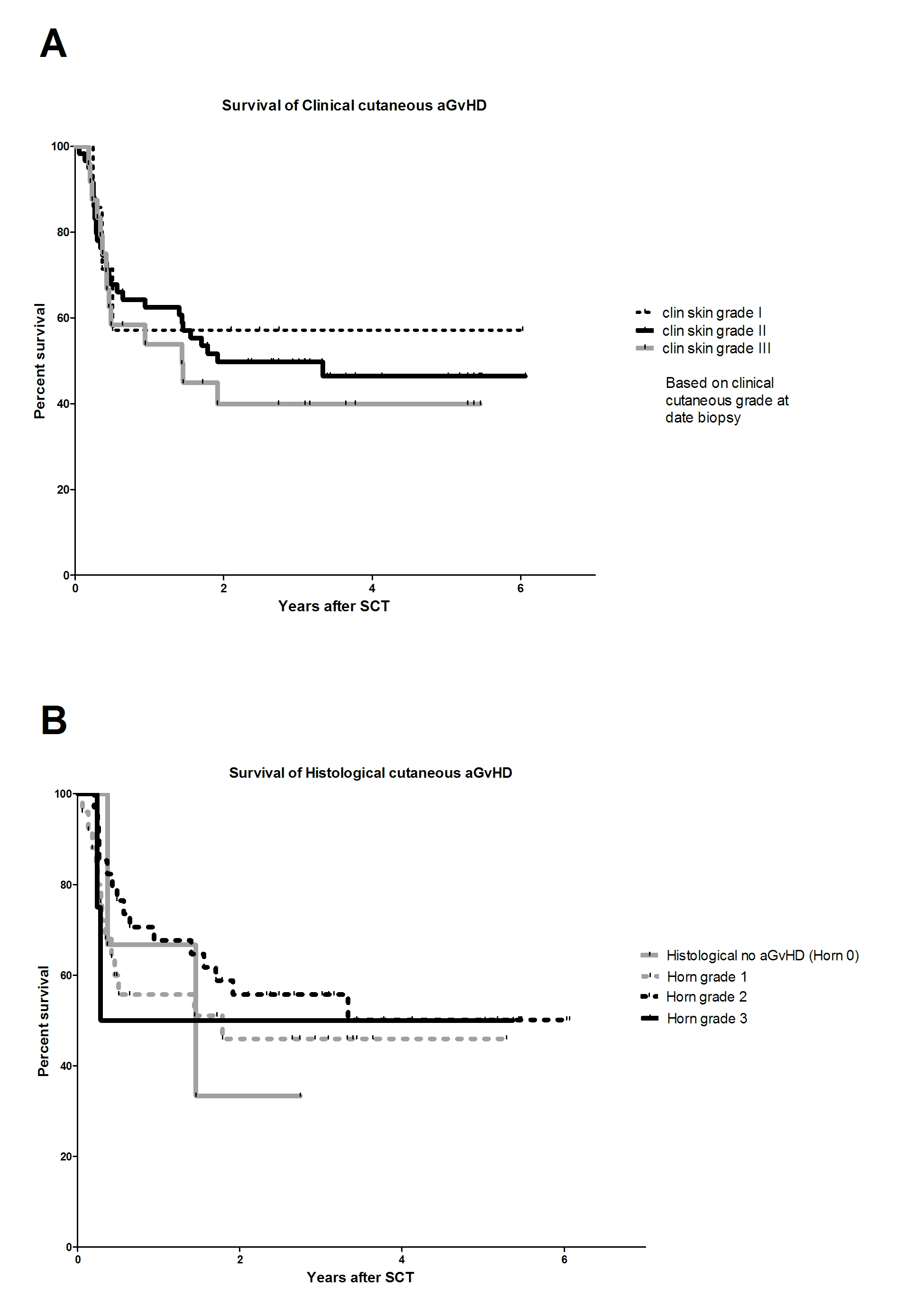

Supplement: Supplementary file 1 — Additional file 1: Figure S1. Survival proportions of acute cutaneous GvHD in patients presenting as acute GvHD at time biopsy (n = 66). A. Survival comparison of acute clinical cutaneous GvHD grading (p = 0.6812). B. Survival comparison of histologically acute GvHD grade in skin biopsies (p = 0.8111). [file 40001_2019_377_MOESM1_ESM.tif]

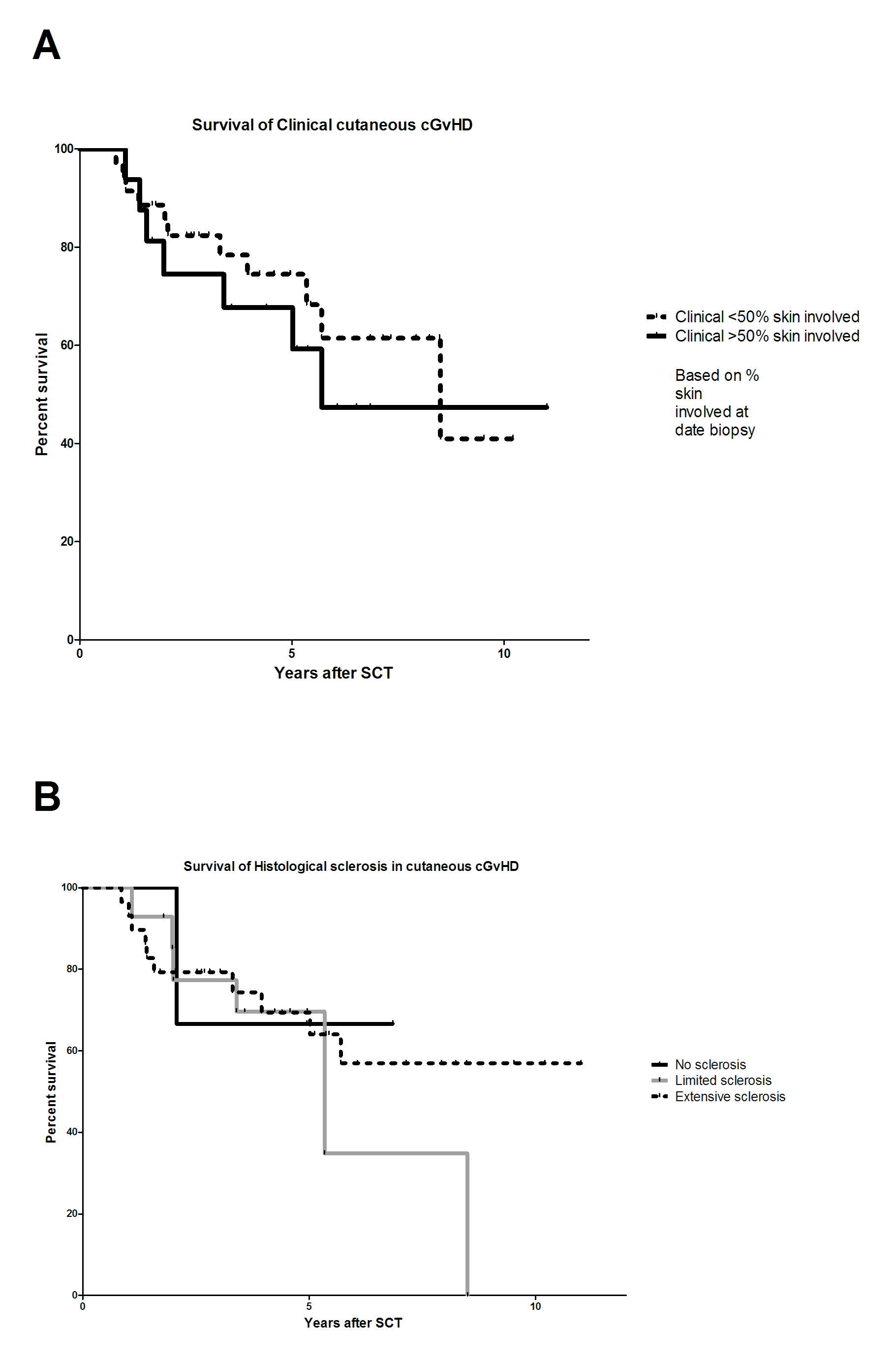

Supplement: Supplementary file 2 — Additional file 2: Figure S2. Survival proportions of chronic cutaneous GvHD in patients presenting as chronic GvHD at time of biopsy (n = 48). A. Survival comparison between clinical percentage of skin involved (p = 0.5231). B. Survival comparison of histologically chronic GvHD grade in skin biopsies (p = 0.7996). [file 40001_2019_377_MOESM2_ESM.tif]
